# Addressing environment, social and governance (ESG) investment in China: Does board composition and financing decision matter?

**DOI:** 10.1016/j.heliyon.2024.e30783

**Published:** 2024-05-07

**Authors:** Naiping Zhu, Ernest Nii Teiko Aryee, Andrew Osei Agyemang, Ishmael Wiredu, Abdulrasheed Zakari, Samuel Yayra Agbadzidah

**Affiliations:** a School of Finance and Economics, Jiangsu University, Zhenjiang, 212013, China; b School of Business, University of Wollongong, Australia; c Alma Mater Europaea -ECM, Slovenska ulica 17, 2000 Maribor, Slovenia; d School of Management, Wuhan Textile University, Wuhan, 430200, China

**Keywords:** Board composition, ESG investment, Financing decisions, Manufacturing firms, China

## Abstract

This study examined the link between board composition and environment, social and governance (ESG) investment, and how financing decisions moderate this nexus. The study constructed hypotheses using insights derived from stakeholder and agency theories. We used secondary data from 2010 to 2022 to conduct an empirical analysis using the system Generalized Method of Moments (GMM) and Fixed Effect (FE) estimators. This study found a positive and significant relationship between board independence, sustainability committee, gender diversity, managerial ownership, board meetings and ESG investment. We also found a negative connection between CEO duality, board size, foreign nationals on the board, annual remuneration, and ESG investment. Furthermore, financing decisions significantly moderated the relationship between board composition and ESG investment. The results confirm the importance of board composition and financing decisions in ESG investment in Chinese manufacturing firms. The results show that splitting the CEO and chairperson roles and frequent board meetings can improve a company's ESG investment. Policymakers should facilitate company operations by providing regulations for ESG investment.

## 1 Introduction

The recent years of accelerated industrialization and urbanization in China have stained the country's efforts to safeguard the environment, maintain social stability, and conserve natural resources [1]. Businesses are under increasing pressure from stakeholders to disclose more information about the beneficial and detrimental impacts of their social and environmental actions and how they integrate environmentally friendly practices into plans and operation models [2]. Firms have paid more attention to Environmental, Social, and Governance (ESG) performance. ESG performance has substantially contributed to business growth and societal development by assessing a company's ecological and social responsibility and governance [3]. According to Ref. [4], ESG performance is now a critical measure of non-financial success, managerial skill and risk mitigation. Therefore, firms must include specific environmental and social policies in their activities. ESG has become an institutionalized approach that has emerged to satisfy the information demands of investors and other stakeholders regarding the ecological, social, and governance impacts of a business' activities [5].

Investors are placing a premium on ESG information to value investment and evaluate the risk of listed firms [6]. ESG investment is a tenet that urges firms and investors to consider environmental impact, social accountability, and corporate governance when making strategic decisions [7]. More businesses are incorporating ESG goals into their investment strategies. In this regard, financial institutions have shown interest in the environmental and social effects and advocated to align with the United Nations Sustainable Development Goals [8]. Stock markets and regulators are all contributing to help ESG investing reach the next efficiency level. Financing decisions and other sustainability-related factors are now essential in light of the societal and environmental shifts [9]. [10] discovered a significant relationship between ESG performance and firm financial performance. According to Ref. [11], institutional investors benefit A-listed companies in China, which is more pronounced for companies with higher expectations for ESG performance. ESG performance enhances company investment and establishes a fundamental corporate strategy [12]. ESG performance reduced financial risk in China during COVID-19 [13].

A company's long-term success requires good corporate governance practices, including effective allocation of funds and retaining and expanding capital. Board attributes have been recognized as influencing environmental and social performance [14]. According to Ref. [15], better ESG policies are linked to sound corporate governance because they help firms establish positive connections with their stakeholders, improving their public reputation and market share [16]. [17] reported that board members influence corporate social responsibility, and for that reason, board members must steer the company in the direction of environmental sustainability and social responsibility.

In contrast to the numerous studies on corporate governance and ESG performance, corporate governance and ESG investment have yet to receive much attention [14,[18], [19], [20], [21]]. investigated the relationship between corporate governance and ESG performance. According to their research, corporate governance mechanisms improve ESG performance. No prior study focused on corporate governance and ESG investment, although ESG investment is gaining traction globally due to its potential for significant environmental and social issues. While it is encouraging to see a movement toward more ethical and environmentally friendly methods, it is necessary to know whether or not the corporate governance attributes enhance ESG investment as interest in ESG investing grows in the international setting.

Therefore, this study aims to investigate the impact of board composition on ESG investment and further seeks to examine the moderating role of financing decisions on board composition and ESG investment nexus.

By drawing insights from stakeholder theory [22] and agency theory [23], we highlighted the significance of good governance attributes in ESG investment. The board should develop ESG investment strategies that serve the interests of stakeholders. From the stakeholders' perspective, the board of directors is appointed to serve the interests of various stakeholders. Therefore, they are responsible for making decisions regarding ESG investment. Board members are in a position to hold management accountable for being transparent regarding ESG performance and investment decisions. Boards are encouraged to incorporate ESG investment into business objectives to align with social and environmental directives to create a sustainable impact for all parties involved. Moreover, the impact of board composition on ESG investment was explained using agency theory. The reason is that there are fewer information disparities between management and stakeholders when a well-structured board is in place [23]. This, in turn, improves corporations' reporting of financial and non-financial environmental and social activities. A fundamental tenet of agency theory is the need to reduce conflict of interest by bringing together the interests of stakeholders, board and management. The board ensures that management serves the interests of its shareholders by prioritizing wealth maximization and sustainable initiatives. Board members with knowledge and experience in social and environmental matters effectively monitor management execution of ESG investments.

In terms of novelty, this study focused on how board composition influences ESG investment decisions rather than just how corporate governance impacts ESG performance. ESG investment is essential for businesses, the economy and societies, and it is worthwhile to investigate the connection between board composition and ESG investments. This study considers boards' independence, diversity, and process and adds knowledge to ESG investment in China. Second, the study incorporates financing decisions as a moderating variable. This study sheds light on the intricate relationship between board composition, sustainable practices, and financial strategies by investigating how ESG investment is impacted by economic factors interacting with board composition. Third, as China has been transitioning towards more sustainable practices, the study developed an index and initiatives to guide companies' ESG practices to promote sustainability goals. The study proposed a new index for quantifying ESG investment based on Sustainable Fitch's ESG ratings. This index combines ESG and sustainable investment measurement for a more robust understanding. Incorporating this index broadens the ESG index in literature and leads to compelling practical and policy implications. Fourth, this study is more relevant to firms, investors, and policymakers because it integrates stakeholder and agency theories. It aims to promote responsible financial management, strengthen stakeholder involvement and help firms create sustainable value.

This study makes significant contributions to the limited existing empirical investigations. First, while most prior studies concentrated on corporate governance and ESG performance, we broadened the scope of the existing studies on ESG by investigating the connection between board composition and ESG investment. This study contributes to literature perspectives on the governance elements that influence ESG investment and the moderating impact of financing decisions on the relationship. The study focused on the financial factors of ESG and provides insights into how robust governance frameworks encourage businesses to invest in ESG to promote sustainable investment and moral trade on a global scale. Second, this study contributes to theoretical knowledge of sustainable investment decisions by integrating stakeholder and agency theories. This study provides a more detailed analysis of mechanisms behind corporate governance, environmental policies, and financial factors by combining various theoretical viewpoints. The concepts offer a more thorough approach to satisfying the needs of different stakeholders, reducing agency problems in corporate settings and understanding the dynamics of sustainable financing decisions. Third, this study provides insights to policymakers in formulating regulations and standards that encourage firms to consider ESG investments to serve stakeholders’ interests. This study contributes to the establishment of regulations which foster corporate responsibility.

The outline for the rest of the paper is as follows. The next section reviews relevant literature on ESG investment and board composition. The study's hypotheses and the broader theoretical context on which it is based are stated. The methodology of the study is discussed in detail in section 3. The data analysis and discussion of the primary results are presented in section 4. The final part of the paper discusses key findings, implications, and recommendations for further research are presented in section 5.

## 2 Literature review

### 2.1 Theoretical concept

#### 2.1.1 Stakeholder theory

Based on the principle of stakeholders, a corporation cannot just focus on its owners' needs if it wants to thrive. According to the stakeholder theory, a company's long-term performance depends on how well it handles its relations with its stakeholders [24,25]. A business is successful if it meets the needs of all of its constituents. Given the context of this investigation, stakeholder theory, which stresses the importance of addressing ESG issues, becomes relevant. By considering ESG investment, businesses can enhance their reputation, lessen potential threats, and attract ethical investors [26]. Financial decisions are crucial to improving resource use, openness, and stakeholder engagement.

According to the stakeholder theory, a firm's ESG efforts can positively affect its bottom line. When upper management is happy in their positions, the company performs better financially and can last long in the market [27]. Corporate governance favours ESG investment, as ESG performance can help solve disagreements between management and stakeholders [15]. Enforcing ESG practice is crucial to preserving and growing a company's bottom line and its worth to shareholders.

Based on the stakeholder perspective, ESG investment policy boosts a company's investment efficacy since stakeholders are more likely to be supportive [28]. According to Ref. [29], firms with excellent ESG investment ratings are more likely to prioritize future financial results because directors are bound to deeper inquiry and evaluation from a broader range of stakeholders, increasing their motivation to embark on initiatives with a favourable net present value.

#### 2.1.2 Agency theory

The agency theory posits that when management is distinct from ownership, there is room for disarray of interests due to managers' varying dedication objects, risk preferences, and length of time frontiers [19,30]. Good corporate governance attributes are crucial accountability tools that safeguard the interests of shareholders, and agency expenses must be kept to a minimum [31]. Management is deterred from advantageously concealing information and is prompted to give more financial and non-financial openness under the strict oversight setting assured by board members [32].

Based on the agency theory perspective, corporations with sound corporate governance are more likely to acknowledge the credibility gap and stakeholders' ESG interests in their business reporting [33]. Having a transparent atmosphere in which management is incentivized to improve the transmission of ESG information to the public is primarily the result of the qualities of the board [34]. Using the framework of agency theory [23], one may claim that managers and shareholders have an agency dilemma when implementing ESG investments. In this view, investing in ESG initiatives is counterproductive because it reduces profit.

Potential manifestations of agency problems arise when it comes to ESG investment. Managers may invest in ESG for their advantage or excessively invest in ESG to get private gains, such as enhancing their reputation at owners' expense [35]. It is argued that companies involved in ESG investment should spend their money wisely. In addition, to fund ESG activities, businesses may have to forego other lucrative endeavours [35]. ESG investment is seen to drain a company's resources, making it less competitive with less conscious rivals. Moreover, the management pragmatism argument states that managers invest in ESG activities using the business fund to deflect blame and explain poor financial decisions. Goodwill publicity is sought through ESG investment to mask poor performance [36].

### 2.2 Hypothesis development

#### 2.2.1 Board independence and ESG investment

According to agency theory, independent directors provide the mechanisms for checks required to minimize information disparity and ameliorate agency disputes [20]. Non-executive directors are more motivated to exercise productive and unbiased oversight of management decisions to safeguard their reputations [37]. From the agency theory perspective, independent directors are more inclined to prevent managers from engaging in opportunistic investment [17]. The reason is that having non-executive board members makes the board more effective and restricts management from acting opportunistically. Since the non-executive members' existence indicates openness and the absence of collusion with management, it is essential to prevent managers from investing in ESG for their self-interest. Directors' fees are the only financial interest non-executive directors have; thus, they are more inclined to curb ESG investment opportunities.

Drawing from the stakeholder theory, independent board members can pressure and demand management to disclose ESG information [18]. Non-executive directors significantly impact ESG disclosure to serve stakeholders' interests [20]. We argue that independent directors influence ESG investment decisions since non-executive directors are tasked with safeguarding the welfare of stakeholders, ensuring transparency and complying with legislation. In light of this, we assume that independent directors foster better investment decisions.

H1 A positive and significant relationship exists between board independence and ESG investment.

#### 2.2.2 CEO duality and ESG investment

According to agency theory, good corporate governance should have checks and balances, but when one person holds a CEO/Chairmanship position, it leads to an imbalance in authority [19]. We argue that less openness and accountability results from CEO duality, which diminishes independence and ability to oversee managerial actions. According to Ref. [38], CEO duopoly steers the board's decisions to their benefit, prioritizing investments that maximize short-term profits at the expense of other stakeholders. This, in turn, decreased disclosure and increased information asymmetry in ESG. Based on this, we argue that CEO duality contradicts the stakeholder theory perspective, which highlights the need to consider the interests of all parties involved. According to Ref. [39], one person occupying the CEO and board chair position decreases ESG performance [15]. reported that CEO duality negatively influences ESG disclosure. The negative connection aligns with the theoretical and empirical justification. This leads us to propose the following hypothesis.

H2 CEO duality is negatively connected with ESG investment.

#### 2.2.3 Board size and ESG investment

Under the stakeholder theory, larger boards pressure managers to adopt practices that align with shareholders and investors [40]. [19] found that larger board sizes are more likely to pressure management to increase transparency and align their actions with stakeholders. Having more board members improves a firm's ESG disclosure, which helps safeguard the firm's legitimacy [14]. From the agency theory perspective, a large board size promotes more excellent supervision [23]. Furthermore, the larger board size may be better equipped with oversight due to the knowledge and experience held by the board members. Firms with many directors tend to make environmental and social disclosures [41]. In contrast, a large board makes it harder for directors to communicate and coordinate, reducing the efficiency of oversight duties and slowing down investment decision-making [32]. reported that boards with fewer members were better at deterring managers from engaging in advantageous conduct and achieving consensus decisions. This promotes greater openness and responsibility concerning ESG investment.

From the tenet of agency theory, we argue that a large board size makes it harder to reach an agreement on a firm's investment decisions. In contrast, from the stakeholder point of view, a large board size can pressure management to release more details about their environment and social efforts and investment decisions to boost their image. In light of this substantial theoretical and empirical backing, the subsequent hypothesis is proposed.

H3 Board size positively and significantly influence ESG investment.

#### 2.2.4 Sustainability committee and ESG investment

Based on the viewpoint of stakeholder theory, a sustainability committee represents an outlook toward meeting those stakeholders' requirements and positively contributing to ESG disclosure [42]. A dedicated sustainability committee aids the board in methodologically outlining and assessing operational actions, policies, and strategies about all aspects of the firm's environmental and social sustainability. Sustainability committees influence ESG disclosure and enhance financial performance [43]. Such a committee also plays a crucial role in ensuring that ESG reporting is accurate and transparent [44]. Accordingly, a sustainability committee is vital to ensure that the firm's operations are legitimate, in line with societal standards, and that various interested parties' demands are acknowledged [32]. In light of the importance of ESG considerations, it is recommended that companies establish sustainability committees. Companies can improve accountability and openness regarding their ESG investment by forming a dedicated sustainability committee. Therefore, such a committee is essential for ESG investment. Based on the theoretical standpoint, we propose that.

H4 Sustainability committee has positive and significant effect on ESG investment.

#### 2.2.5 Gender diversity and ESG investment

Agency theorists argue that boards should strive for gender diversity because men and women are culturally, socially and personally distinct [19]. A more balanced representation of women on corporate boards improves managerial oversight and independence [45]. This, in turn, encourages more openness and responsibility regarding financial and non-financial environment and social matters. From the tenet of stakeholder theory, having female directors allows for a broader representation of stakeholders [3]. It is argued that participation from women board members will help meet the information demands of various stakeholder groups. Female representation on board is often seen as an indication of conformity to market and environmental standards [39]. [45] found that female directors influence a firm's disclosure of ESG. Therefore, we argue that women board members can make significant contributions to decisions involving ESG investment due to their diverse backgrounds. Based on this solid theoretical backing, we proposed that.

H5 Gender diversity has positive and significant effect on ESG investment.

#### 2.2.6 Foreign nationals and ESG investment

From the agency theory perspective, the presence of international directors in the boardroom strengthens oversight, leading to better tactical choices about economic and social initiatives and improved reporting on those operations [46]. Appointing a board member from various countries, as advocated by Ref. [20], boosts board effectiveness because it allows corporations to benefit from the global perspective and expertise of their directors in addressing ESG matters. Companies need the flexibility to adapt their operations to new financial markets and stakeholder preferences to stay competitive.

Appointing directors from various cultural backgrounds has been shown to boost ESG performance by companies [46]. [47] argue that foreign nationals are valuable business resources that enhance a company's competitive edge and ESG performance. ESG reporting is bolstered when foreign nationals with worldwide experience and expertise are appointed to boards. Based on this theoretical and empirical basis, we hypothesized that.

H6 There is a positive and significant connection between foreign nationals and ESG investment.

#### 2.2.7 Managerial ownership and *ESG investment*

There are two competing theories regarding managerial ownership and ESG. According to the agency theory, when there is high managerial ownership, managers are likely to make short-term decisions to protect their interests rather than the stakeholders’ [48], which raises agency costs. This implies that managerial owners are more likely to invest in environmental and social activities that will benefit them and adversely affect the company in the long run. According to Ref. [47], managers have more leeway to act arbitrarily without facing consequences when there is high managerial ownership.

In contrast, from a stakeholder point of view, there is a better chance that management and stakeholders’ interests will be aligned when there is a higher level of managerial ownership [49]. Based on this, we argue that managers with shares execute outstanding ESG activities, which boosts the firm reputation and, in turn, increases the value of the firm. Environmental and social responsibilities are considered when there is a greater degree of managerial ownership [50], which brings the management and stakeholders into agreement. Managerial ownership reduces costs and fines associated with environmental damage. We argue that the cost associated with environmental damage influences managerial ownership to invest in ESG.

H7 Managerial ownership positively impacts ESG investment.

#### 2.2.8 Annual remuneration and *ESG investment*

From the stakeholder theory perspective, boards with more independence and financial incentives are more inclined to render unbiased decisions by advocating for more transparency [31]. In addition, boards are under stakeholders' pressure to reveal more information about executive remuneration, which is understandable given that board members have a deeper grasp of the company's inner workings and want to explain their roles. According to Ref. [51], directors prioritizing ESG receive lower salaries and participate in environmental and social responsibilities to improve the company's performance and align with stakeholders' interests. This implies that directors who are more forthcoming about their ecological and governance practices take a pay cut. Boards are concerned about this trend and demand further information to elucidate the principles behind this practice. Board members' compensation is a likely factor in the financial decision to provide information voluntarily. According to Ref. [31], a board's remuneration increases the likelihood of making fair financial decisions by advocating for greater ESG disclosure. Therefore, it is reasonable to presume that the directors' remuneration impacts ESG investment.

H8 There is a positive and significant relationship between annual remuneration and ESG investment.

#### 2.2.9 Board meetings and *ESG investment*

Agency theory suggests that increasing board meetings can narrow shareholder-management informational gaps and the associated agency expenditure [31]. The regular board meetings also facilitated the free flow of information between the board and executive leadership. From the stakeholder theory perspective, board meetings allow board members to share information and views to make better decisions to guarantee that all stakeholders' demands are served. According to Ref. [52], significant environmental and social issues are addressed during regular board meetings, which serve as an essential arena for decision-making. Board members can effectively oversee and disclose firm's environmental activities and reap benefits for stakeholders when the board meets frequently [14]. It is argued that frequent board meetings are a valuable resource for enhancing ESG performance. Regular meetings are required to organize activities deemed beneficial to ESG investment. Therefore, regular board meetings would be necessary if the board considers ESG investment decisions alongside those on regular business operations.

H9 Board meeting is positively and significantly related to ESG investment.

#### 2.2.10 The moderating role of financing decisions on board composition and ESG investment nexus

Consistent with stakeholder and agency theories, firms must consider and safeguard stakeholders' interests, decreasing information asymmetry within stakeholders. According to the stakeholder theory perspective, companies should consider stakeholders' interests when financing investment objectives to ensure the investment is ethical [53]. Companies that rely on equity funding are in a better position to balance the needs of their stakeholders since they are less financially leveraged and less obligated to debt [54]. In contrast to the stakeholder theory perspective, debt holders put a premium on financial gains and risk reduction, which could restrict a company's capacity to serve the interests of different stakeholders. Companies prioritizing environmental and social aspects reaped the benefits of stable equity funding [55].

The agency theory is consistent with governance attributes, asserting that better governance reduces agency conflict, which lowers agency costs and levels of debt [56]. Lenders are likelier to provide lower interest rates to firms that disclose their ESG practices [57]. Furthermore, highly leveraged companies protect their shareholders’ interests and reduce the likelihood of bankruptcy by implementing ESG strategies [56]. They further stated that companies prioritizing social and environmental responsibility have lower debt-to-equity ratios.

Thus, consistent with stakeholder theory, it is reasonable to assume that companies that use much equity finance will have a favourable connection between firm governance and ESG investment. The reason is that firms can better prioritize the interest of a broader range of stakeholders when they use equity financing for ESG investment. We argue that companies that rely heavily on debt funding must put short-term profits ahead of long-term sustainability goals. This leads us to postulate the following.

H10 Financing decisions substantially moderates the board composition and ESG investment nexus.

## 3 Methodology

### 3.1 Sampling technique and data source

This study utilized a quantitative research approach to investigate the relationship between board composition and ESG investment in Chinese A-listed manufacturing companies. The manufacturing industry is known for its substantial environmental and social impacts. This sector often faces ESG-related issues, including resource management, pollution control, and labour conditions. We obtained secondary data from the firm's annual report on the China Stock Market Accounting Research (CSMAR) database from 2010 to 2022. For several reasons, we settled on the 2010 to 2022 period as the study's time frame. China underwent improvements in firm governance and financial structure after introducing the Chinese Accounting Standards (CAS) in 2007. Also, firms began to provide ESG-related data after 2009 in China [56]. The timeframe allows for robust statistical analysis and reliable findings because it covers the COVID-19 period. Purposive sampling was employed to select the 248 manufacturing companies. The study considered 132 manufacturing firms from the Shanghai Stock Exchange and 116 from the Shenzhen Stock Exchange. The sample size was determined based on data availability and represented the sample in the broader population of Chinese manufacturing companies.

### 3.2 Measurement of variables

#### 3.2.1 Dependent variables

##### 3.2.1.1 ESG investment index

We used a content analysis strategy to determine the dependent variable. We adapted the ESG investment index from Eikon Refinitiv, MSCI and Sustainable Fitch's ESG investment rating. ESG investment index was employed to assess a firm's investment in ESG. There are three primary themes under the ESG investment index: investment in environment, social, and governance. There are five specific indicators under each category. A score of 1 is given if the corporation disclosed the investment information and a score of 0 otherwise. The details of the scoring are presented in Table 1.

**Table 1:** ESG investment index.

Themes	Code	Indicator details
Environmental Investment	EI1	Funding for environmentally friendly research and development,
	EI2	Funding for product-eco-design and eco-product innovation policies
	EI3	Funding for training on environmental management and development
	EI4	Funding for environmental preservation and policy
	EI5	Funding for safeguarding the environment.
Social Investment	SI1	Investments in employee safety and development
	SI2	Community engagement funding
	SI3	Investments in society development projects
	SI4	Corporate social responsibility funding
	SI5	Investments in labour practice and training
Governance Investment	GI1	Funding governance structures
	GI2	Establishing laws on ethical behaviour
	GI3	Determining how much top executives are paid
	GI4	Funding of shareholders' share rights
	GI5	Funding stakeholders' participation

We utilized the unweighted scoring technique by dividing the actual disclosure score by the maximum score possible as expressed in Equation (1).

(Equation 1) $$ESGI=\frac{Actual\;ESG\;investment\;score}{Total\;expected\;ESG\;investment\;score}$$

#### 3.2.2 Independent variables

Board composition is the independent variable for the study. We divided the board characteristics into five attributes: independence, structure, diversity, incentives, and process. We established the following variables under each attribute: Board independence, CEO duality, board size, sustainability committee, board gender diversity, foreign nationals, managerial ownership, annual remuneration, and board meeting.

#### 3.2.3 Moderating variable

We used financing decisions as the moderating variable for the study. Equity and debt financing were used as proxies to measure financing decisions. Data for financing decisions is extracted from firms' annual reports from 2010 to 2022.

#### 3.2.4 Control variables

We also include profitability, leverage, and firm size as control factors to remove potential bias. Extensive research on the characteristics and aspects related to board attributes and ESG investment informed the selection of these control variables. More profitable firms are believed to implement better ESG policies [3]. Profitability is proxied as Return on equity (ROE). Also [58], reported that a firm with higher leverage is associated with improved environmental and social outcomes. Leverage is measured as total debt to total assets. Finally, the size of a firm is a crucial indicator of its CSR performance [49]. We measured firm size as the natural logarithm of total assets. Table 2 presents the summary of the study variables.

**Table 2:** Summary of study's variables.

Category	Board composition	Variables	Symbol	Measurement
Dependent variable		ESG investment	ESGI	Dividing the actual ESG investment disclosure score by the total possible disclosure score
Independent variables	Independence	Board independence	BI	Proxied by number of non-executive directors on the board
		CEO duality	CD	1 is given if the same person is CEO and chairperson at a time and 0 for otherwise
	Structure	Board size	BS	Proxied by the number of board of directors
		Sustainability committee	SC	1 is given if the firm has sustainability-related committee; otherwise, 0
	Diversity	Board gender diversity	GD	Number of female directors on the board
		Foreign nationals	FN	Measured by the number of foreign directors on the board
	Incentives	Managerial ownership	MO	Measured by percentage of shares held by executive directors and management
		Annual remuneration	AR	Proxied by total remuneration paid to board members
	Process	Board meetings	BM	Number of times the board members met during the year
Moderating variable	Financing decision	Debt financing	DF	Percentage of total debt to total financing
		Equity financing	EF	Percentage of total equity to total financing
Control variables		Return on Equity	ROE	Earnings after tax and interest divided by equity
		Leverage	LEV	Total debt divided by total assets
		Firm size	FSZ	Proxied by natural logarithm of total assets

### 3.3 Model specification

We construct the following panel regression model to analyze the effect of board composition on ESG investment for hypotheses 1 to 9 as shown in Equation (2).

(Equation 2) $${ESGI}_{it}=\beta_{0}+\lambda {ESGI}_{it-1}+\beta_{2}{BI}_{it}+\beta_{3}{CD}_{it}+\beta_{4}{BS}_{it}+\beta_{5}{SC}_{it}+{\beta_{6}{GD}_{it}+\beta_{7}{FN}_{it}+\beta }_{8}{MO}_{it}+\beta_{9}{AR}_{it}+\beta_{10}{BM}_{it}+\beta_{11}ROE+\beta_{12}LEV+\beta_{13}FSZ+\varepsilon_{it}$$

Our final hypothesis (H10) relates to the moderating effect of financing decisions on the link between board composition and ESG investment. We generated two equations for the financing decision.

Debt financing (DF) moderates the relationship between board composition and ESG investment, as shown in Equation (3).

(Equation 3) $${ESGI}_{it}=\beta_{0}+\lambda {ESGI}_{it-1}+\beta_{2}{\left(BI\times DF\right)}_{it}+\beta_{3}{\left(CD\times DF\right)}_{it}+\beta_{4}{\left(BS\times DF\right)}_{it}+\beta_{5}{\left(SC\times DF\right)}_{it}+{\beta_{6}{\left(GD\times DF\right)}_{it}+\beta_{7}{\left(FN\times DF\right)}_{it}+\beta }_{8}{\left(MO\times DF\right)}_{it}+\beta_{9}{\left(AR\times DF\right)}_{it}+\beta_{10}{\left(BM\times DF\right)}_{it}+\beta_{11}ROE+\beta_{12}LEV+\beta_{13}FSZ+\varepsilon_{it\;}$$

As shown in Equation (4), equity financing (EF) moderates board composition and ESG investment.

(Equation 4) $${ESGI}_{it}=\beta_{0}+\lambda {ESGI}_{it-1}+\beta_{2}{\left(BI\times EF\right)}_{it}+\beta_{3}{\left(CD\times EF\right)}_{it}+\beta_{4}{\left(BS\times EF\right)}_{it}+\beta_{5}{\left(SC\times EF\right)}_{it}+{\beta_{6}{\left(GD\times EF\right)}_{it}+\beta_{7}{\left(FN\times EF\right)}_{it}+\beta }_{8}{\left(MO\times EF\right)}_{it}+\beta_{9}{\left(AR\times EF\right)}_{it}+\beta_{10}{\left(BM\times EF\right)}_{it}+\beta_{11}ROE+\beta_{12}LEV+\beta_{13}FSZ+\varepsilon_{it}$$

Where ESGI represents ESG investments, BI symbolizes board independence, CD indicates duality CEO duality, BS is board size, SC stands for sustainability committee, GD is board gender diversity, FN denotes foreign nationals, MO indicates managerial ownership, AR represents annual remuneration and BM indicates board meetings. Debt financing (DF) and Equity (EF) financing are the moderating variables. ROE denotes return on equity, LEV stands for leverage, and FSZ indicates firm size as the control variables. $\beta_{0}$ for the constant term, ε for the error term, i and t for chosen company and years, respectively.

### 3.4 Data processing

We present a brief overview of the methods used in this study, including the Friedman cross-sectional dependency test, Westlund cointegration test, Generalized Method of Moments (GMM) and Fixed Effect (FE) estimators. Friedman cross-sectional dependency test identifies problems related to temporal dependency undermining independent presumptions required for accurate inference. A cointegration test was performed to investigate the persistence of the relationship between variables. GMM was employed as the primary estimator, and the static Fixed Effect (FE) estimator was chosen as the starting point based on the preliminary analyses' findings. These estimators are robust to distributional constraints and used to fix unobserved heterogeneity, endogeneity biases and serial association of the study's variables.

## 4 Results and discussion

### 4.1 Cross-sectional dependence test

When analyzing data panels, it is essential to account for cross-sectional dependence, which occurs when variables within the same cross-section are linked because of shared, unobserved causes. This is recognized as a ubiquitous cross-sectional dependency, and it can affect analysis reliability by introducing biases [54]. The level and amount of cross-sectional dependency can be assessed by considering the degree and type of cross-sectional linkages within the variables. Imprecise estimates can emerge from overlooking cross-sectional dependence, which could undermine the validity of the findings. We run the Friedman test to evaluate cross-sectional dependence, as shown in Table 3.

**Table 3:** Friedman cross-sectional dependency result.

Test	Statistics	P-value
Friedman (FE)	18.462	0.0024
Friedman (RE)	15.563	0.0078

In Table 3, cross-sectional dependence in the research factors is indicated by the p-values of 0.0024 and 0.0078 for the fixed effect (FE) and random effect (RE), respectively. The values were statistically significant for both FE and RE at a 1 % level. The results show that there is substantial evidence that there is cross-sectional dependence, which contradicts the null hypothesis. The results show that the observation in the data is significantly correlated across different time points. The findings imply that a shock in one manufacturing firm would affect the other.

### 4.2 Cointegration test

The cointegration test is an essential technique in analysis to evaluate whether a set of non-stationary variables share a long-run relationship. It is necessary to do the cointegration test to check the long-term linkages between the study's variables because the absence of one shows that they do not have such a connection [59]. Long-term associations between variables were verified using the Westerlund test. We opted for the Westlund test because it can sidestep cross-sectional dependence problems and endogeneity-related dissatisfaction [60]. The null hypothesis in the Westerlund test is that no cointegration exists among the variables. The Westerlund cointegration outcome is shown in Table 4.

**Table 4:** Westerlund cointegration test result.

	Statistics	P-value
Variance ratio	3.3462	0.0005

Table 4 shows that the independent variables were significantly linked to the dependent variable over the long term. A p-value of less than 0.1 implies a long-term connection between the variables. As a result, we conclude that there is cointegration between the research variables and reject the null hypothesis that there is no cointegration. The cointegration test helps to eliminate the possibility of erroneous estimates. As a result, we will use that estimate in our research.

### 4.3 Estimation techniques

We utilized the system Generalized Method of Moments (GMM) estimator as the main estimator and the static Fixed Effect (FE) estimator as the starting point. The GMM estimator solves the issue of autocorrelation and heteroskedasticity [27]. GMM handles hidden standard shocks to avoid endogeneity problems caused by inverse causality and biased selection [61]. The fixed effect estimator enables heterogeneity to be accounted [62].

Panel A comprises manufacturing companies that are traded on the Shanghai Stock Exchange. Panel B features publicly traded companies on the Shenzhen Stock Exchange. Panel A and B were merged and regressed into Panel C. The estimated association between board composition and ESG investments in China is displayed in Table 5 below.

**Table 5:** Results of multiple regression analysis of the relationship between board composition and ESG investment.

Variables	Panel A		Panel B		Panel C	
	FE	GMM	FE	GMM	FE	GMM
ESGI_(it-1)_		0.408***		0.324***		0.520***
		(0.019)		(0.013)		(0.023)
lnBI	0.025**	0.103***	0.032*	0.094**	0.047**	0.128***
	(0.003)	(0.006)	(0.011)	(0.010)	(0.005)	(0.007)
lnCD	−0.012	−0.034*	0.018*	−0.029*	−0.028*	−0.047**
	(0.015)	(0.014)	(0.006)	(0.009)	(0.010)	(0.003)
lnBS	−0.008*	−0.015**	0.005	−0.009*	0.016*	−0.024**
	(0.002)	(0.009)	(0.005)	(0.003)	(0.005)	(0.004)
lnSC	0.042**	0.076*	0.038*	0.061**	0.073*	0.146**
	(0.003)	(0.012)	(0.014)	(0.005)	(0.026)	(0.011)
lnGD	0.018*	0.052***	0.015**	0.048*	0.032*	0.112***
	(0.005)	(0.002)	(0.001)	(0.013)	(0.011)	(0.004)
lnFN	−0.030	−0.061	−0.025	−0.054	−0.065	−0.134
	(0.025)	(0.053)	(0.024)	(0.043)	(0.062)	(0.012)
lnMO	0.035**	0.032**	0.030	0.028*	0.065	0.057*
	(0.002)	(0.004)	(0.023)	(0.005)	(0.041)	(0.010)
lnAR	−0.022	−0.016	−0.020	−0.016	−0.045	−0.039
	(0.015)	(0.012)	(0.019)	(0.014)	(0.031)	(0.029)
lnBM	0.009*	0.011**	0.006	0.011*	0.024*	0.032**
	(0.002)	(0.007)	(0.004)	(0.001)	(0.003)	(0.006)
lnROA	0.071* (0.017)	0.062** (0.005)	0.048 (0.047)	0.036* (0.009)	0.094** (0.010)	0.082** (0.006)
lnLEV	−0.102 (0.084)	0.092* (0.022)	−0.072* (0.013)	0.064* (0.019)	0.112 (0.106)	0.108* (0.026)
lnFS	0.048* (0.015)	0.041* (0.013)	0.051** (0.005)	0.038* (0.012)	0.071** (0.007)	0.620* (0.077)
Firm	Yes	Yes	Yes	Yes	Yes	Yes
Year	Yes	Yes	Yes	Yes	Yes	Yes
Adjusted R squared	0.625		0.538	Adjusted R squared	0.712	
AR(1) (z, Pr > z)		−2.89(0.310)		−3.34(0.253)		−2.41(0.364)
AR(1) (z, Pr > z)		−1.61(0.601)		−2.01(0.482)		−1.12(0.684)
Hansen Test (chi2, Pr > z)		28.23(0.625)		34.02(0.512)		19.45(0.731)
Sargan Test		918.09		728.94		1024.28
Fisher Statistics		13,012.87		9184.01		15,628.25
Prob > Chi2		0.000		0.000		0.000
**Observation**	**1584**	**1520**	**1392**	**1352**	**2976**	**2872**

Note: Standard errors are in parenthesis.

***, **, * represents 1 %, 5 % and 10 %, respectively.

The result in Table 5 indicates that the first order of autocorrelation does not exhibit any signs of first-order autocorrelations. The probability values for AR(1) and AR(2) reveal this to be true. In addition, the results of the Hansen and Sargan tests show that the alternative hypothesis is rejected, and the null hypothesis of exogeneity is accepted. This means that the estimators' results are accurate and reliable.

The results of the main estimator in Table 5 reveal a positive and statistically significant relationship between board independence and ESG investment in all panels. These findings suggest that increased board independence corresponds to a positive and noteworthy uptick in ESG investment. This implies that independent board members play a pivotal role in shaping corporate decisions prioritizing sustainability, environmental responsibility, and social impact, underling the importance of strong governance in fostering ESG commitment within Chinese manufacturing companies.

A negative and statistically significant relationship exists between CEO duality and ESG investment in all panels. This suggests that when the CEO holds both the CEO and board chair positions, there is a notable decrease in ESG investment. The results imply that the board's capacity to adequately oversee and coordinate the firm's social and environmental efforts can be impaired when CEO dualism exists. These insights underscore the importance of considering governance reforms to separate the responsibility of CEO and board chair. Separating this role improves independence and oversight, leading to firm decision-making that involves long-term investment and environmental responsibility.

Board size reveals a positive and statistically significant negative relationship in Panel C. This indicates that a percentage increase in board size will result in a decrease in ESG investment. Thus, the larger boards are associated with a notable reduction in ESG investment. The result suggests that a larger board size can hinder decision-making and reaching an agreement on ESG matters. Strategic plans, specifically those about ESG investing, can face drawn-out debates and standstill due to the increased diversity of viewpoints that comes with a larger board. These findings suggest that larger boards allocate fewer resources to ESG investment. The discovery highlights the significance of carefully considering board size.

The sustainability committee positively and significantly associates ESG investment across all panels. This indicates that a percentage increase in sustainability committees among firms' boards results in significant ESG investment. The result signifies that firms with dedicated sustainability committees demonstrate a pronounced commitment to an upswing in ESG investment. The firm's sustainability efforts must be directed and monitored by sustainability committees. Such committees ensure that ESG elements are part of strategic decisions by focusing on sustainability-related issues and allocating resources accordingly. Firms should establish sustainability committees to guarantee that ESG activities align with firm strategy and contribute to sustainable development.

Gender diversity revealed a positive and statistically significant connection with ESG investment. This underscores that a percentage change in female board members demonstrates a pronounced commitment to ESG investment. Female directors actively propel ESG investment decisions, creating an environment that prioritizes environmental and social responsibility and sustainability impact. Gender diversity enhances board deliberation, ultimately leading to holistic consideration of ESG investment.

Foreign nationals posit an adverse and insignificant link between ESG investment in Panel A, B, and C. These findings imply that a unit decrease in foreign directors on board will not significantly negatively influence ESG investment within Chinese manufacturing companies. Board members from other countries enrich the board with cultural insights and experience. However, differences in conventions and regulatory frameworks can exacerbate cultural disparities, making it harder to comprehend and prioritize ESG investment. The cultural distinctions can hinder effective communication and decision-making towards ESG matters, leading to adverse ESG investment. The consistent trend across all panels prompts further exploration into the complex dynamics surrounding foreign board members and their impact on ESG investment strategies.

Managerial ownership signifies a positive and significant relationship between ESG investment in all panels. This implies that firms where managers have a substantial ownership stake demonstrate a pronounced commitment to ESG investment. Managerial owners tend to consider long-term initiatives and channel resources toward ESG activities to foster firm sustainability. Firms can design incentive structures that reward directors for ESG performance that aligns their interest with the firm's sustainability goals.

Table 5 reveals that annual remuneration has a negative and insignificant relationship with ESG investment in all panels. This implies that a percentage decrease in directors' yearly remuneration will not negatively impact ESG investment. The negative trend across all panels warrants further investigation into the dynamics between directors' compensation and the impact on ESG investment. The results suggest that directors may focus on initiatives that bring immediate financial gains, including cost reduction, instead of investing in ESG activities. Firms should evaluate and adjust Directors' remuneration plans to incorporate ESG performance indicators.

Board meetings and ESG investment revealed a positive and statistically significant association in all panels. The positive and significant relationship suggests that firms conducting more frequent board meetings tend to increase ESG investment. The results indicate that board members can discuss and deliberate on ESG-related investment, including strategies and risk management. This result reaffirms the role of frequent board meetings in promoting ESG investment, emphasizing the board's influence in fostering sustainability practices. Firms should hold frequent board meetings to deliberate on ESG investment decisions.

Finally, the starting static fixed effect results are remarkably comparable to the main estimator (GMM) results in Table 5. We conclude that GMM results in Table 5 are reliable. Hence, policymakers can rely on the findings.

### 4.4 Moderating analysis

The study looked at how financing decisions affected the ESG investment of manufacturing firms listed on the Shanghai and Shenzhen Stock Exchanges in China. The results of the interaction between the board attributes and financing decisions are presented in Table 6. The table also contains the interaction between board attributes, debt financing, and equity financing. A-listed manufacturing firms listed in both the Shanghai Stock and Shenzhen Stock Exchange were aggregated in the testing. Each section underwent a pair of tests. The moderating effect of debt financing on both board composition and ESG investment was tested using Fixed Effect and GMM. We used the exact estimators to test for the moderating effect of equity financing.

**Table 6:** The moderating role of financing decision on board composition and ESG investment.

Variables	Debt financing		Variables	Equity financing	
	FE	GMM		FE	GMM
ESGI_(it-1)_		0.491***	ESGI_(it-1)_		0.482***
		(0.020)			(0.025)
lnBI × DF	0.125* (0.024)		**BI**×**EF**	0.134** (0.009)	
		0.092** (0.011)			0.087* (0.021)
lnCD × DF	−0.076 (0.074)		**CD**×**EF**	0.101* (0.032)	
		−0.098* (0.016)			0.113** (0.010)
lnBS × DF	0.115** (0.014)		**BS**×**EF**	0.129* (0.051)	
		0.104** (0.006)			0.091 (0.081)
lnSC × DF	0.107** (0.008)		**SC**×**EF**	0.121** (0.011)	
		0.095* (0.031)			0.089** (0.011)
lnGD × DF	0.112 (0.103)		**GD**×**EF**	0.128** (0.016)	
		0.115 (0.113)			0.087* (0.021)
lnFN × DF	0.102* (0.024)		**FN**×**EF**	0.117 (0.096)	
		0.118** (0.012)			−0.103* (0.034)
lnMO × DF	0.125** (0.013)		**MO**×**EF**	0.131* (0.021)	
		0.103** (0.010)			0.096* (0.027)
lnAR × DF	−0.106** (0.008)		**AR**×**EF**	0.113* (0.028)	
		−0.088* (0.017)			0.101** (0.012)
lnBM × DF	0.114* (0.035)		**BM**×**EF**	0.127 (0.110)	
		0.097** (0.006)			0.084* (0.018)
Control variables	Yes	Yes	Control variables	Yes	Yes
Firm	Yes	Yes	Firm	Yes	Yes
Year	Yes	Yes	Year	Yes	Yes
Adjusted R squared	0.725		Adjusted R squared	0.794	
AR(1) (z, Pr > z)		−2.13(0.382)	AR(1) (z, Pr > z)		−3.09(0.214)
AR(1) (z, Pr > z)		−1.26(0.521)	AR(1) (z, Pr > z)		−1.94(0.625)
Hansen Test (chi2, Pr > z)		29.34(0.631)	Hansen Test (chi2, Pr > z)		35.42(0.724)
Sargan Test		851.37	Sargan Test		942.26
Fisher Statistics		19,034.43	Fisher Statistics		23,157.21
Prob > Chi2		0.000	Prob > Chi2		0.000
**Observation**	**2976**	**2872**	**Observation**	**2976**	**2872**

Note: Standard errors are in parenthesis.

***, **, * represents 1 %, 5 % and 10 %, respectively.

The results of the main estimator (GMM) in Table 6 are very similar to the FE values. Therefore, we interpret the results of the main estimator (GMM).

Table 6 results from the Sargan test show that the method effectively considers financing decisions' role in moderating the board composition and ESG investment of Chinese manufacturing firms. There is also substantial variety in the moderating variable's impact on the dependent and independent variables, as seen by the AR (2) values.

The result indicates a positive and statistically significant connection between board independence and debt financing in influencing ESG investment. This implies that the presence of non-executive directors has a more beneficial effect on a company's commitment to ESG investment when the company relies on debt funding. Robust governance systems may raise ESG investment since debtholders value them to protect their interests. **Similarly**, there is a positive and statistically significant interaction between board independence and equity financing in influencing ESG investment. Including non-executives increases a company's dedication to ESG investment when using equity funding. According to the findings, the relationship between board independence and ESG investment is significantly influenced by both debt and equity financing.

The interaction between CEO duality and debt financing has a negative and statistically relationship with ESG investment. This suggests that when there is CEO duality and firms rely on debt financing, ESG investment is negatively affected. This is because CEOs prefer to use equity finance rather than debt finance, though equity finance is expensive than debt finance. Debt holders may perceive CEO duality as a governance concern that detracts from ESG investment. **However**, there is a positive and statistically significant relationship exists between CEO duality and equity financing and ESG investment. In highly equity-funded firms, CEO duality shows commitment and enhance ESG investment. CEOs prefer equity financing for investment because of no interest payment which means lower bankruptcy risk.

Board size and debt financing is positively and significantly associated with ESG investment. Debt financing amplifies the positive relationship between boar size and ESG investment, likely due to debt holders' preference for strong governance and risk management practices. **Similarly**, equity financing positively moderates the connection between board size and ESG investment. Equity investors often appreciate diverse and well-governed boards that align with ESG goals. A larger board size positively influences ESG investment, and debt and equity funding improve this influence.

The interaction between sustainability committee and debt financing is positively and significantly influence ESG investment. This discovery highlights the significance of sustainability as a mechanism for encouraging ESG investment. Debt holders place a premium on sustainability committees to gauge a firm's commitment to sustainable practices and sound financial management, which in turn encourages more investment in ESG. **Like debt financing**, equity financing moderates the positive relationship between sustainability committees and ESG investment. There is more dedication to ESG investments in equity-funded firms with a sustainability committee because equity financing enhances firm flexibility as compared to debt financing. The sustainability committee shows that business is serious about ESG and fiscal responsibility.

The interaction between gender diversity and debt financing on ESG investment is positive but the relationship is insignificant. The result suggests that debt financing does not significantly improve the connection between gender diversity and ESG investment. This implies that the beneficial effect of gender diversity on ESG remains regardless of the debt financing. Firms with more female board members tend to prioritize sustainability regardless of the financing decision. **In contrast**, when firms rely on equity funding, gender diversity has a positive and statistically significant impact on ESG investments. This implies that female representation on boards is mainly influential in ESG investment when firms rely on equity funding. The result suggests that gender diverse boards are better able to deploy resources toward ESG because equity financing is free from debtholders pressure to prioritize financial gain.

There is a positive and significant relationship between foreign nationals-debt financing interaction and ESG investment. The result indicates that debt financing strengthens the relationship between foreign directors and ESG investments. This suggests that, when using debt funding, the presence of foreign nationals is associated with increased ESG investment commitment. **Contrarily**, when firms rely on equity funding, foreign nationals have an adverse effect on ESG investment. This implies that the beneficial impact of foreign directors on ESG investment is reduced due to shareholder demand for short-term financial gain.

The interaction between managerial ownership and debt financing has positive and substantial impact on ESG investment. This indicates that when firms rely on debt financing, ESG activities are given more priority with managers with significant ownership stakes. Debtholders’ pressure to firms to demonstrate sustainability motivate managerial owners to invest in ESG. **Similarly**, there is a positive and statistically significant relationship between managerial ownership and ESG investment using equity financing. Equity financing provides firms with long-term funding and flexibility, enabling managerial owners to invest in ESG activities without short-term pressure from debtholders. Firms can use these insights to consider the benefits of higher managerial ownership levels in fostering ESG activities and responsible financial management practices.

Annual remuneration and debt financing have a negative and **significant** relationship with ESG investment. This indicates that higher annual remuneration associated with reduced ESG investment is weakened by debt financing. When a firm has a debt leverage with high annual remuneration of directors' result decreased in ESG investment. **Conversely**, the interaction between annual remuneration and equity financing is statistically significant and positively associated with ESG investment. This implies that equity financing strengthens the impact of annual remuneration on ESG investment. This result reveals that directors' annual remuneration influence firms ESG investment when they rely on equity finance. The finding suggests that with equity funding, executive compensation is tied to the firm's ability to generate value for shareholders and other stakeholders. Consequently, directors may be motivated to prioritize ESG activities that boosts the firm's growth and image in the long run.

Finally, a positive and statistically significant connection between board meetings and debt financing impacts ESG investment. Firms with active and engaged boards are more inclined to prioritize and commit resources to ESG investment, mainly when they rely on debt funding. This reflects a dedication to responsible financial management practices and aligns with the preference of debt holders. **Similarly**, a significant positive association exists between board meetings and equity financing in impacting ESG investment in all panels. Frequent board meetings signal a commitment to ESG investment. These findings highlight the influential role of board meetings in promoting ESG investment, regardless of the financing decision method chosen by firms.

### 4.5 Discussion

Investment in ESG has become increasingly important in modern business. Due to the potential for long-term value creation and risk avoidance, investors are increasingly looking to align their portfolios with companies that commit to ESG investment. This discussion aims to clarify the mechanisms underpinning the link between board composition and ESG investments and elaborate on the far-reaching ramifications of these significant results.

First, board independence is a pillar of the governance system and acts in the stakeholders' interest in the firm's decision-making process [20]. An independent board of directors is critical in determining how a company handles ESG investment. Based on our findings, board independence is positive and significantly relates to ESG investment, hence accepting H1. The positive and substantial effect of board independence on ESG investment indicates the crucial role that non-executive members offer in a firm's environmental and social investment. From the tenet of agency theory, non-executive directors are required to perform their duty with due diligence in the best interest of their shareholders and other stakeholders. Independent directors reduce agency problems and improve accountability. The results align with [63], emphasizing that independent directors are more inclined to consider all stakeholders' needs and include ESG factors in the firm's overall strategy. Consistent with the stakeholder perspective, independent boards can offer strategic supervision required to ensure that a company's ESG investments are not just temporary but also part of its long-term strategy.

In addition, when one person performs CEO and chairmanship responsibilities, there may be less autonomous monitoring and a diminished ability to analyze management choices, especially those relating to ESG issues [69]. We found a negative and significant relationship between CEO duality and ESG investment; therefore, H2 is accepted. The result indicates that CEO duopoly can impair the prioritizing of ESG activities within the firm. The result supports the findings of [15], who found that CEO dualism have adverse impact on ESG performance. From the tenet of agency theory, separating the positions of CEO and board chair increases independence, minimizing the risk of management encroachment and increasing accountability to stakeholders. From the stakeholders' standpoint, the division of responsibilities between the CEO and board chair sends a message about the importance of good governance and increasing environmental and social investment.

Furthermore, our research shows that board size negatively links ESG investment. Based on this finding, H3 is rejected. The finding suggests that board size can have difficulties in making decisions and reaching agreement on social and environmental matters. The findings disagree with [14], who reported that board size positively influence environmental disclosure. While many board members may have more diverse viewpoints and specializations, there may be challenges in coordinating and making decisions efficiently, which can slow down the growth of ESG investment. From the standpoint of stakeholder and agency theories, a small board size can better make decisions leading to ESG activities to serve stakeholders' interests [32]. Boards with fewer members are more likely to be nimble and effective in their discussions, which could allow them to respond better to ESG investment challenges and opportunities.

Several companies have established sustainability committees inside their boards to address ESG issues as a concrete demonstration of their dedication to ethical business practices [44]. This aligns with stakeholder theory, suggesting that sustainability committees foster open communication and accountability by including various interested parties. The study discovered that the presence of a sustainability committee significantly influences ESG investment; therefore, we accept H4. The result indicates that a sustainability committee improves a firm's standing on ESG investment. The finding corroborates with [43], who reported that sustainability committees integrate ESG into long-term decision-making, aligning with various stakeholders' interests.

Gender diversity on corporate boards enhances board independence and managerial supervision. Women board members demonstrate the firm's dedication to environmental and social justice, boosting its credibility and appeal to financiers and other stakeholders. Our research shows that gender diversity positively and significantly impacts ESG investment; hence, we accept H5. The result implies that the firm's actions align with ESG investing due to women's board representation. This result aligns with stakeholder and agency theories, which emphasize the need to meet the demands and interests of a wide range of stakeholders. ESG-oriented investors are drawn to companies with a high proportion of women serving in leadership roles because it shows they care about ethical business practices and accountability [33]. The study supports the findings of [3] that women directors enhance social and environmental performance.

Moreover, incorporating the viewpoints and experiences of foreign nationals enriches decision-making on ESG performance [47]. Company policies on ESG investing may change depending on the nationalities of board members. Our study found that foreign nationals on corporate boards have a negative and insignificant effect on ESG investment; hence, we reject H6. The result implies that the presence of foreign nationals on Chinese manufacturing boards does not automatically lead to significant changes in ESG investment decisions. The result disagree with [46], who reported that foreign directors enhance ESG performance. Although a diverse board can better address ESG matters, foreign nationals have the potential to cause priority misalignment due to cultural complexities, making it harder to work together on ESG investment.

Our research highlights the significance of managerial ownership structure in determining a firm's ESG investing strategy. We found a positive and significant relationship between managerial ownership and ESG investment, thereby accepting H7. The finding shows that managers who are shareholders are more inclined to prioritisze ESG goals over immediate financial gains. This aligns with agency theory because managerial ownership aligns with shareholder and management interests, thereby decreasing agency problems. This congruence with the stakeholder theory perspective showing managerial ownership shows dedication to the firm's long-term social and environmental performance, boosting the trust of stakeholders [49].

Executive salaries, bonuses, and other kinds of annual remuneration are significant factors in corporate governance and executive decision-making [51]. Our research shows a negative relationship between annual compensation and ESG investment; hence, we reject H8. The negative connection suggests high annual remuneration can redirect funds from ESG investment. The finding resonates with agency theory, highlighting that directors with high annual remuneration may be motivated to prioritize short-term profits at the expense of ESG investment, leading to agency problems [31]. Reevaluating annual remuneration can be necessary for sustainability-focused firms to guarantee that remunerations align with ESG investment and stakeholders’ interests.

Effective corporate governance relies on board meetings, facilitating decision-making, oversight, and long-term planning [14]. This shows that board meetings are critical for influencing a business' ESG investment strategy. Our investigation reveals the positive and significant connection between board meetings and ESG investment. Therefore, we accept H9. The result supports the findings of [52]. The beneficial impact highlights that directors will have more time to evaluate and discuss ESG investment at more frequent board meetings. This supports agency theory, which emphasizes the board members' task in overseeing the best interest of shareholders. Furthermore, from the stakeholder perspective, board meetings and members' participation in ESG investment signal commitment to environmental and social matters. Boards should meet often to offer a formal framework for in-depth talks on ESG investment.

Firms' sustainability in ESG activities depends on their approach to funding. The stakeholder theory suggests that to accomplish ESG investment, businesses should carefully control their financing decisions [53]. Firms can improve their ESG investment by making better financing decisions that lead to sustainable development. A firm's financing decision should be carefully thought out and handled if it wants to expand and strengthen its ESG investment. This study discovered a favourable moderating effect of financing decisions on board composition and ESG investment. This leads to accepting H10. Nevertheless, some of the board attributes have a negative and insignificant effect on ESG investments when moderated with debt and equity financing. From the shareholders' perspective, equity financing for ESG investment could be more favourable because raising equity financing will increase the business's capital cost, leading to agency problems. It is suggested that firms consider the benefits of regular and engaged board discussions on sustainability matters. This can help set ESG investment priorities, monitor progress, and demonstrate commitment to stakeholders. The study's findings are similar to Ref. [54], who found a positive link between financing decisions and ESG performance.

## 5 Conclusion and policy implications and limitations

### 5.1 Conclusion

This study examined how board composition influences ESG investment in Chinese manufacturing firms. In addition, the study further examined whether financing decisions moderate this relationship. This research applies stakeholder and agency theories to show how board governance impacts ESG investment decisions. Businesses that invest in their ESG activities decrease information disparities with investors and other stakeholders, ease funding limitations and boost investment capacity by being more transparent. This will help align with stakeholders’ interests.

The study used a quantitative approach and relied on data from 2010 to 2022. We employed Fixed Effect and GMM estimators for the regression analysis. The results show that board independence, sustainability committee, gender diversity, managerial ownership, and board meetings impacted ESG investments. However, CEO duality, foreign nationals, and annual remuneration adversely affect ESG investment. We also found that financing decisions moderate the connection between board composition and ESG investment.

Companies should include more non-executive directors and women on their boards to enhance ESG investment and performance. Establishing and enhancing sustainability committees is also recommended for firms wanting to improve their ESG performance and engage responsible investors. Businesses should encourage top executives to hold sizeable shares to improve ESG investment. Additionally, policymakers and industry associations should support responsible governance practices facilitating ESG investment.

The study contributes to literature by investigating the connection between board composition and ESG investment within the setting of Chinese A-listed manufacturing companies. In addition, this research adds to the body of knowledge in corporate finance and governance by illuminating the moderating role of financing decisions in the connection between board composition and ESG investment. Lastly, this study sheds light on the difficulties developing-world businesses encounters in balancing financial and ESG performance. Policymakers should make it a top priority to incorporate ESG policies into corporate settings.

### 5.2 Policy implications

Policymakers will benefit from the result of this study by creating several rules and regulations on corporate governance and ESG concerns. First, the study will help policymakers develop and implement effective governance and sustainability policies. It can help shape policy changes to promote ESG integration and ethical finance practices in the business world. Policymakers are urged to assist businesses by providing greater motivation for investment in ESG. In addition, this study provides evidence to policymakers to guide companies in implementing ESG principles. This study can help policymakers adjust ESG policies to align with sustainable decisions. The study will help the government fulfil its orienting role, promote ESG practices, and ensure economic development. Furthermore, authorities should compel firms to disclose and make public information regarding substantial ESG-related investments. Also, there must be training programmes for businesses to address concerns in their ESG plans. Lastly, this study can help governments and businesses create more environmentally friendly industries. In a more practical sense, ESG investment can improve corporate performance by assisting firms in continuing sustainable practices, building trust with stakeholders, raising their reputation, and helping solve global sustainable development problems. Moreover, the result can help firms adjust their governance system to better ESG targets. This study will help managers create and apply ESG strategies to lessen the impact of information asymmetry and the resulting inefficiencies in ESG investments.

### 5.3 Limitations

A few caveats to this study will need to be addressed in future studies. First, the study's sample is limited to Chinese manufacturing firms; future research should broaden its scope to include firms from industries and other geographical regions. In addition, demographic variables, including educational level, knowledge, age, and behavioural traits, were not considered when analysing the effect of board composition on ESG investment. Future studies can look at these characteristics of the board. Lastly, the study did not provide alternative measures for ESG investment. Future researchers should consider alternate ways to measure ESG investment.

## Data availability

Secondary data were extracted from the sample firm's annual reports and financial statements. Majority of the data was sourced from the China Stock Market Accounting Research (CSMAR) database from 2010 to 2022.

## Funding

This research was supported by the 10.13039/501100012456National Social Science Foundation of China (Grant No. 20BGL099).

## CRediT authorship contribution statement

**Naiping Zhu:** Writing – review & editing, Writing – original draft, Funding acquisition, Formal analysis, Data curation, Conceptualization. **Ernest Nii Teiko Aryee:** Writing – review & editing, Writing – original draft, Methodology, Formal analysis, Data curation, Conceptualization. **Andrew Osei Agyemang:** Writing – review & editing, Writing – original draft, Methodology, Formal analysis, Data curation, Conceptualization. **Ishmael Wiredu:** Writing – review & editing, Writing – original draft, Methodology, Formal analysis, Data curation, Conceptualization. **Abdulrasheed Zakari:** Writing – review & editing, Writing – original draft, Methodology, Formal analysis, Data curation, Conceptualization. **Samuel Yayra Agbadzidah:** Writing – review & editing, Writing – original draft, Methodology, Formal analysis, Conceptualization.

## Declaration of competing interest

The authors declare that they have no known competing financial interests or personal relationships that could have appeared to influence the work reported in this paper.
